# Human cerebrospinal fluid contains diverse lipoprotein subspecies enriched in proteins implicated in central nervous system health

**DOI:** 10.1126/sciadv.adi5571

**Published:** 2023-08-30

**Authors:** Nathaniel J. Merrill, W. Sean Davidson, Yi He, Ivo Díaz Ludovico, Snigdha Sarkar, Madelyn R. Berger, Jason E. McDermott, Linda J. Van Eldik, Donna M. Wilcock, Matthew E. Monroe, Jennifer E. Kyle, Kimberley D. Bruce, Jay W. Heinecke, Tomas Vaisar, Jacob Raber, Joseph F. Quinn, John T. Melchior

**Affiliations:** ^1^ Biological Sciences Division, Pacific Northwest National Laboratory, Richland, WA 99354, USA.; ^2^ Center for Lipid and Arteriosclerosis Science, Department of Pathology and Laboratory Medicine, University of Cincinnati, Cincinnati, OH 45237, USA.; ^3^ Department of Medicine, University of Washington School of Medicine, Seattle, WA 98109, USA.; ^4^ Department of Molecular Microbiology and Immunology, Oregon Health and Science University, Portland, OR 97239, USA.; ^5^ Sanders-Brown Center on Aging, University of Kentucky, Lexington, KY 40504, USA.; ^6^ Division of Endocrinology, Metabolism and Diabetes, School of Medicine, University of Colorado Anschutz Medical Campus, Aurora, CO 80045, USA.; ^7^ Department of Neurology, Oregon Health and Science University, Portland, OR 97239, USA.; ^8^ Division of Neuroscience, Department of Behavioral Neuroscience and Radiation Medicine, ONPRC, Oregon Health and Science University, Portland, OR 97239, USA.; ^9^ Department of Neurology and Parkinson’s Disease Research Education and Clinical Care Center (PADRECC), VA Portland Healthcare System, Portland OR 97239, USA.

## Abstract

Lipoproteins in cerebrospinal fluid (CSF) of the central nervous system (CNS) resemble plasma high-density lipoproteins (HDLs), which are a compositionally and structurally diverse spectrum of nanoparticles with pleiotropic functionality. Whether CSF lipoproteins (CSF-Lps) exhibit similar heterogeneity is poorly understood because they are present at 100-fold lower concentrations than plasma HDL. To investigate the diversity of CSF-Lps, we developed a sensitive fluorescent technology to characterize lipoprotein subspecies in small volumes of human CSF. We identified 10 distinctly sized populations of CSF-Lps, most of which were larger than plasma HDL. Mass spectrometric analysis identified 303 proteins across the populations, over half of which have not been reported in plasma HDL. Computational analysis revealed that CSF-Lps are enriched in proteins important for wound healing, inflammation, immune response, and both neuron generation and development. Network analysis indicated that different subpopulations of CSF-Lps contain unique combinations of these proteins. Our study demonstrates that CSF-Lp subspecies likely exist that contain compositional signatures related to CNS health.

## Teaser

The brain generates unique lipid-protein nanoparticles that contain cargo important for maintaining healthy cognitive function.

## INTRODUCTION

Plasma and cerebrospinal fluid (CSF) contain nano-sized lipid-protein complexes called lipoproteins that are best recognized for their role in transporting lipids to cells and tissues to maintain whole-body lipid homeostasis. Plasma lipoproteins are well studied and characterized, being traditionally classified on the basis of particle density into chylomicrons, very-low-density (VLDL), low-density (LDL), and high-density (HDL) lipoproteins. LDL is commonly referred to as “bad cholesterol” and HDL as “good cholesterol” because of their opposite associations with cardiovascular disease. However, lipoproteins are more compositionally complex than simple cholesterol; they contain a neutral lipid core of cholesteryl ester and triglycerides that are surrounded by a monolayer of phospholipid interspersed with free cholesterol. These lipid emulsions are associated with scaffold proteins called apolipoproteins that provide structural integrity to the particles and act as detergents for transport of the hydrophobic lipid constituents through the aqueous environment. The major scaffold protein for VLDL and LDL is apolipoprotein (*APO*)*B* and for HDL is *APOA1*.

Recent advances in biophysical separation techniques and the advent of high-sensitivity mass spectrometers have revealed substantial compositional heterogeneity within each of the lipoprotein subclasses, particularly for HDL (*1*, *2*). Studies have demonstrated that HDL contains more than 250 different proteins and more than 200 different lipids (*3*). These proteins and lipids coalesce into discrete particles of different sizes (i.e., HDL subspecies) that contain both common and unique complements of proteins that act cooperatively in functions beyond lipid transport ranging from inhibition of proteases to activation of the complement cascade (*4*). Perhaps the most impressive example of HDL’s functional specialization is the trypanosome lytic factor (TLF): an HDL that contains *APOA1*, haptoglobin-related protein (*HRP*), and *APOL1*. TLF leverages *HRP* on the particle surface to infiltrate the parasite *Trypanosoma brucei brucei* through a Trojan horse mechanism. Upon gaining access, *APOL1* undergoes a pH-induced conformational change within the lysosome to form a transmembrane pore resulting in osmotic swelling and lysis of the parasite (*5*). TLF is one of only a handful of specific HDL protein complexes that have been successfully resolved to date (*6*), as similarities in biophysical features across particle populations make them challenging to resolve with singular isolation approaches. Nonetheless, HDL phenotyping studies have now identified unique speciation and compositional signatures related to molecular pathways involved in the innate immune response (*7*), inflammation (*8*), glucose metabolism (*9*), and oxidative stress (*10*), speaking to the pleiotropic functional diversity of HDL. Further, different HDL subspecies differentially track, both positively and negatively, with coronary heart disease (*11*) making them enticing therapeutic targets for treating metabolic disorders (*12*, *13*).

The brain is one of the most lipid-rich organs in the human body, and the central nervous system (CNS) generates its own constellation of lipoproteins that are thought to help maintain CNS lipid homeostasis. Aberrant lipid metabolism in the CNS has now been linked to multiple neurodegenerative disorders including Alzheimer’s disease (AD), Parkinson’s disease, Huntington’s disease, and amyotrophic lateral sclerosis (*14*). Unfortunately, our understanding of these unique particles in the CNS is limited, as they exist in CSF at 1/200th the levels of lipoproteins in plasma (*15*) making detailed biochemical analysis notoriously difficult. Some studies suggest lipoproteins in the CSF (CSF-Lps) are similar to plasma HDL because of shared properties such as size and shape (*15*–*17*). This notion is supported by cursory compositional studies that show that, like HDL, CSF-Lps are primarily enriched in phospholipid and cholesterol (*15*, *16*). In addition, CSF-Lps contain multiple HDL-associated proteins including *APOA1* and *APOE* though unlike plasma HDL, *APOE* is the primary scaffold protein on CSF-Lps (*15*, *17*). Furthermore, HDL-modifying enzymes such as lecithin-cholesterol acyltransferase (*LCAT*) and phospholipid transfer protein (*PLTP*) are present in CSF (*18*) and membrane proteins like adenosine triphosphate–binding cassette subfamily A member 1 (*ABCA1*), essential for HDL biogenesis, are expressed in cells unique to the CNS (*19*). Given the strong similarities between CSF-Lps and plasma HDL, it follows that CSF-Lps may also exhibit HDL compositional heterogeneity and functional pleiotropy.

The revelation on the molecular complexity of HDL is borne out of sophisticated speciation techniques developed to resolve different HDL populations. One informative approach has been high-resolution size exclusion chromatography (SEC) (*2*) that separate particles on the basis of differences in their hydrodynamic diameter. Compared to traditional SEC setups, the integration of three Superdex 200 columns in-tandem vastly broadens the window of elution of particles and proteins in the HDL-size range (*2*). This technique has revealed profound shifts in the HDL speciation profiles of individuals with documented metabolic perturbations, including type 2 diabetes (*20*), obesity (*21*), and pregnancy (*22*). These profiles are typically obtained by measuring lipid abundance (i.e., total cholesterol or phospholipid) across individual fractions using enzymatic assays. Though sufficient for plasma, the application of these assays to human CSF would require upwards of 50 ml of sample (*16*), making analogous studies on CSF-Lps across individuals and thus clinical populations impracticable.

**Fig. 1.**
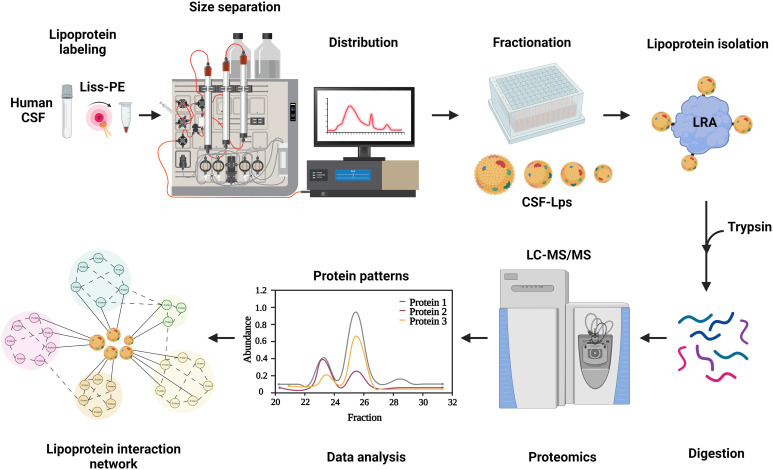
Workflow for molecular characterization of CSF-Lps. Lipoproteins are exchange labeled with a rhodamine-containing phospholipid and size distribution determined using a fluorescent lipoprotein profiler. CSF-Lps within each fraction are isolated using lipid removal agent (LRA). Lipid-bound proteins are digested directly off the resin, collected, and analyzed using bottom-up mass spectrometry (LC-MS/MS). Computational analysis of protein abundance and comigration patterns are used to generate protein network maps and identify potential CSF-Lp subspecies.

In the current study, we used the sensitivity of fluorescence to overcome the lipoprotein abundance obstacle in CSF. Our workflow is illustrated in Fig. 1. We exchange labeled human CSF-Lps with a rhodamine-tagged phospholipid. By engineering an ultrasensitive fluorescent detector into a high-resolution SEC system, we monitored the fluorescent signal of the lipoprotein subspecies as they eluted real time after size separation. Using this fluorescent lipoprotein profiler (FLP), we captured the distribution of lipoproteins in as little as 350 μl of neat CSF. We went on to isolate variously sized CSF-Lp subpopulations and characterize their proteome using liquid chromatography–mass spectrometry (LC-MS/MS). We identified more than 300 proteins associated with CSF-Lps with over half of the proteins unique to the CNS. Last, using a multipronged computational approach, we generated protein network maps across the different particle populations and identified multiple unique protein clusters important for maintaining healthy neurometabolic function.

## RESULTS

### Profiling labeled lipoproteins in plasma

**Fig. 2.**
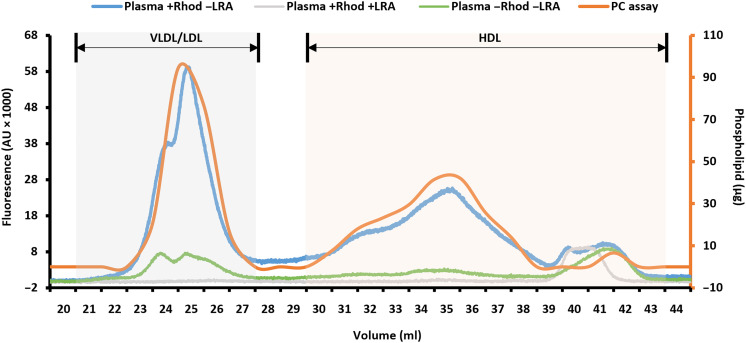
Distribution of lipoproteins in human plasma using FLP. The elution profile of labeled plasma lipoproteins was determined by monitoring rhodamine fluorescent spectra as particles elute from the columns. The distribution of fluorescent signal in labeled plasma is shown in blue. Supernatant collected from labeled plasma that was preincubated with LRA (i.e., lipoprotein depleted) is shown in gray. Autofluorescence of unlabeled plasma is shown in green. Phospholipid distribution was determined enzymatically in individual fractions collected in unlabeled control plasma and shown in orange. The gray area that spans elution volumes 21 to 27 ml is consistent with VLDL/LDL-sized particles, and the light-orange region that spans elution volumes 30 to 43 ml is consistent with HDL-sized particles. AU, arbitrary units; PC, phosphatidylcholine.

Given the size similarities reported between CSF-Lps and plasma HDL (*15*), we reasoned that our SEC method (*20*–*22*) should be a powerful approach for speciating particles in CSF if the sensitivity issue could be overcome. When developing the method, we were concerned that the trace lipid labeling procedure with the Liss Rhodamine phosphoethanolamine (Liss Rhod PE) might (i) artifactually alter lipoprotein size species patterns or (ii) selectively label certain species and not others. To address this, we took advantage of the fact that plasma could be analyzed both with and without label because of its abundance of circulating lipoproteins. Human plasma was labeled and applied to the FLP as described in Materials and Methods. Figure 2 shows the fluorescent signal distribution across plasma lipoproteins (solid blue) versus the distribution of phospholipid in unlabeled plasma from the same individual by traditional enzymatic analysis (solid orange). Elution patterns were generally similar between the samples indicating that the label introduction neither perturbed the lipoprotein size pattern nor selectively labeled certain species. Given the high sampling rate, monitoring the fluorescent signal resulted in improved resolution evidenced by the emergence of a shoulder peak in fractions that corresponded to VLDL/LDL-sized particles that appear as a single peak with the enzymatic measures. As a control, we incubated rhodamine-labeled plasma with lipid removal agent (LRA), which binds phospholipid-associated proteins, before column separation (*2*). Essentially no signal was observed in the LRA supernatant with the exception of a few small fractions indicating minimal nonspecific binding of the Liss Rhod PE to lipid-free proteins in the plasma (gray). Last, we applied unlabeled plasma alone to evaluate interfering autofluorescence of plasma components present in the system (green). While we observed some autofluorescence in the VLDL/LDL-sized fractions, the signal was relatively minor compared to labeled particles (Fig. 2).

Given the sensitivity of fluorescence, the signal intensity can be affected by a number of factors such as labeling volumes, mass, and/or light exposure. To control for these factors, we tested the utility of spiking free rhodamine into our labeling mixture as a loading control. To test nonspecific binding to plasma constituents, we incubated plasma with varying amounts of free rhodamine and evaluated samples using the FLP. As shown in fig. S1A, nonspecific interaction with plasma constituents was not observed at free rhodamine concentrations ≤0.0025 mg/ml. Higher concentrations appeared to increase signal in elution volumes consistent with lipoproteins (fig. S1B); thus, free rhodamine (0.0025 mg/ml) was selected for subsequent studies. In plasma, the free rhodamine eluted in a bimodal fashion spanning a large elution volume range from 85 to 120 ml likely representing a mixture of self-associated and monomeric free rhodamine.

Last, we investigated the relationship between total fluorescent signal and phospholipid mass applied to the FLP. We diluted plasma to different concentrations and then labeled and analyzed the samples using the FLP. As shown in fig. S2A, a decrease in fluorescent signal was observed with decreased mass of plasma phospholipid applied to the FLP. With the exception of the nondiluted sample, the intensity of the free rhodamine peaks was nearly identical across the samples, indicating consistent labeling and sample loading onto the FLP across runs. For diluted samples, we observed only a single free rhodamine peak between 100 and 120 ml (fig. S2B) compared to the undiluted sample. We speculate that this shift reflects a release of self-associated free rhodamine to monomeric form due to the presence of sodium chloride in samples diluted with standard tris buffer (STB). The fluorescent signal abundance revealed an excellent relationship to the mass of phospholipid applied to the FLP (fig. S2C), demonstrating that the FLP is capable of quantifying phospholipid mass in a calibrated system with as little as 3 μg of total phospholipid applied to the columns.

### Profiling labeled lipoproteins in CSF

**Fig. 3.**
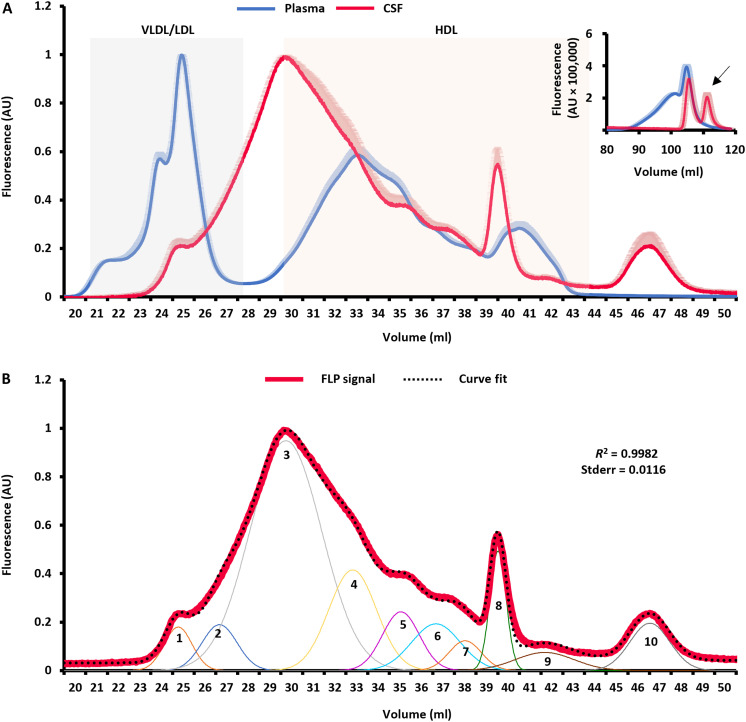
Distribution of lipoproteins in human plasma and CSF using FLP. (**A**) We applied labeled lipoproteins in human plasma (blue) and pooled human CSF (red) to the FLP. The signal is normalized to the most intense peak within respective biofluids for direct comparison. Lines represent the averaged signal across *n* = 5 runs in plasma and *n* = 4 runs in human CSF with light shadowed areas reflecting the SD in signal across replicates. The inset shows signal distribution for the free rhodamine loading control. The “phantom peak” exclusive to human CSF samples elutes at between 110 and 120 ml. We partitioned samples into 1-ml fractions for molecular profiling. (**B**) We analyzed the CSF-Lp elution profile using PeakFit and identified 10 lipoprotein populations of varying concentrations in CSF. The theoretical curve generated by those populations is superimposed on the raw CSF-Lp distribution profile as black dots.

For CSF-Lp studies, we analyzed particles in commercially available pooled human CSF (Medic Biochemica). We applied labeled CSF to the FLP to obtain the elution profile shown in Fig. 3A. The distribution of CSF-Lps (red) showed stark differences compared to plasma lipoproteins (blue). To better delineate particle size distribution, we imported the elution curves into PeakFit for curve peeling analysis (Fig. 3B) which revealed that at least 10 discretely sized populations of lipoproteins exist within the CSF. The bulk of CSF-Lp lipid appeared to elute in a size range between that of plasma VLDL/LDL and large HDL peak (Fig. 3B, peak 3). However, we also observed several smaller CSF-Lp populations in the HDL size range (Fig. 3B, peaks 4 to 7). Notably, we identified two additional peaks that, to our knowledge, have not been previously reported including a large species consistent with VLDL/LDL-sized particles (Fig. 3B, peak 1) and a very small species that eluted downstream of typical plasma HDL (Fig. 3B, peak 10). The variance in signal between the technical replicates (SD ≤8%) indicated excellent reproducibility with labeling and signal detection in both plasma lipoproteins and CSF-Lps. An overlay of individual traces for both human plasma and CSF samples can be found in fig. S3. Curiously, with the CSF samples, we observed an additional peak in the elution volume eluting after the free rhodamine not present in the plasma samples (Fig. 3A).

**Fig. 4.**
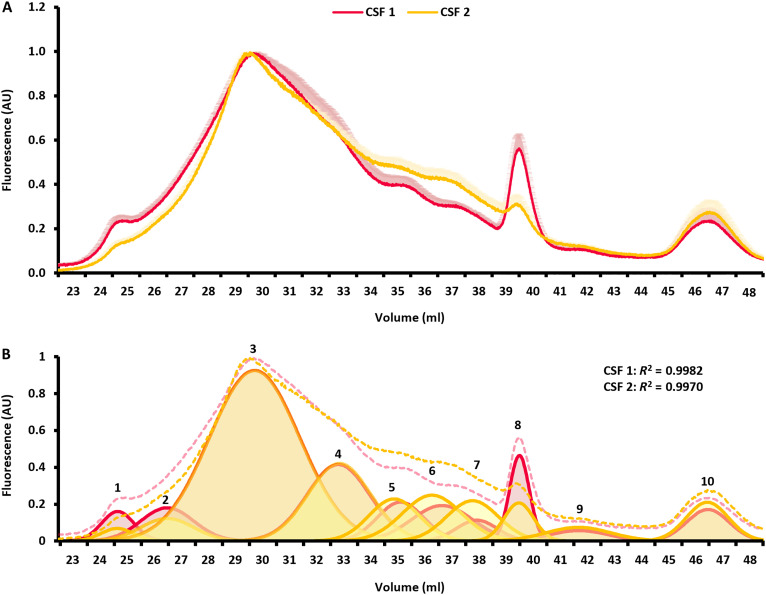
Distribution of lipoproteins in different human CSF samples. (**A**) The FLP signal distribution from labeled lipoprotein in pooled human CSF (red) and CSF collected from a single donor (yellow). The signal is normalized to the most intense peak within runs for direct comparison of subpopulations. Lines represent the averaged signal across *n* = 4 runs in pooled human CSF and *n* = 2 runs in individual human CSF with light shadowed areas reflecting the SD in signal across replicates. (**B**) Quantification of 10 different-sized CSF-Lp subpopulations within each sample using PeakFit. The original curve for each sample is shown as the dashed line and respective subpopulations from PeakFit are shown as colored Gaussian peaks. The *R*^2^ values represent the cumulative fit of the Gaussian peaks to the respective sample traces.

To get a sense of interindividual variation, we labeled and evaluated the CSF-Lp profile in a second CSF sample obtained from a single donor. A comparison of the CSF-Lp profiles between different samples is shown in Fig. 4A. In the single-donor CSF sample, we observed an elevation in several of the HDL-sized regions and a decrease in the larger particles. Nonetheless, we performed a curve peeling analysis (Fig. 4B) that, same as the pooled sample, identified 10 different-sized CSF-Lp populations. Quantification of the individual peaks shows a decrease in peaks 1 and 2 and 8 and increases in peaks 6 and 7 in the individual donor. These results indicate that, like lipoprotein distributions in plasma, differences likely exist in distribution and levels of CSF-Lp subpopulations across individuals.

Last, we ran a series of additional tests to validate our observations in CSF and quantify the impact of sample manipulation on the native CSF-Lp distribuion. We evaluated CSF-Lp distribution using multiple column setups (fig. S4) which all showed the same general elution pattern of the different subpopulations. We confirmed that the highest resolution of CSF-Lps was achieved using the three Superdex 200 columns in series (fig. S4C). As with plasma, autofluorescence was negligible with the exception of a small peak eluting in the size range of albumin (fig. S5A). Though most experiments were run with concentrated CSF, we observed robust signal from as little as 350 μl of native (unconcentrated) CSF (fig. S5B) and observed no major impact on the CSF-Lp elution profile resulting from concentration. Last, we tested the impact of flash freezing on CSF (fig. S5C) and observed minor shifts in the distribution indicating that multiple freeze/thaw cycles could influence the speciation profile.

### Proteomic composition of CSF-Lp subspecies determined by LC-MS/MS

**Fig. 5.**
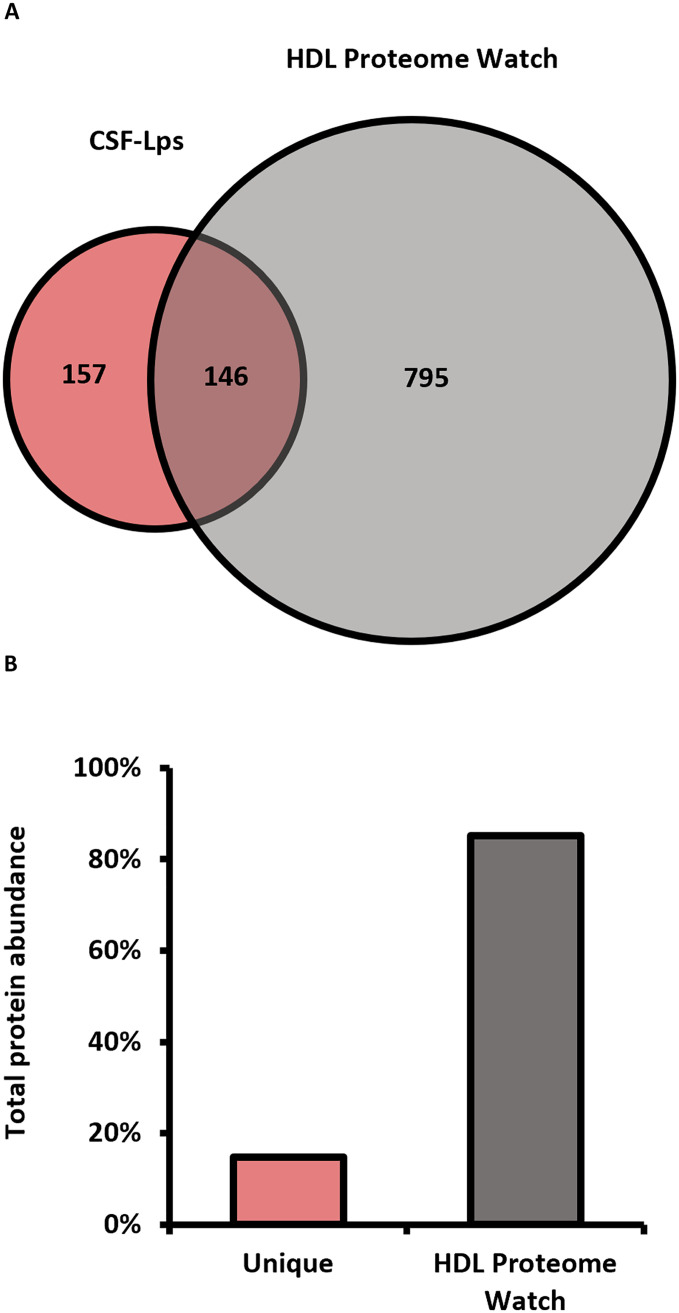
Proteins associated with CSF-Lps compared to the “HDL Proteome Watch.” (**A**) The overlap of proteins identified on human CSF-Lps with proteins reported on the HDL Proteome Watch. The HDL Proteome Watch list only required a single report of a protein on HDL (941 total proteins). (**B**) The relative abundance of the proteins unique to CSF-Lps and proteins overlapping with the HDL Proteome Watch. We calculated abundance by summing LFQ intensities for proteins within each group and expressing individual values as a percentage of total LFQ intensity of all proteins detected across fractions.

We partitioned the CSF from Fig. 3 into 1-ml fractions and isolated lipid-associated proteins away from free plasma proteins within each fraction using LRA (*2*). We digested proteins directly off the lipid-bound particles and collected peptides for analysis by LC-MS/MS. Using MaxQuant, we analyzed the tryptic peptides and identified 303 robust proteins across fractions after filtering as described in Materials and Methods. A full list of the proteins detected and averaged label-free quantification (LFQ) intensities across the three runs can be found in table S1. To evaluate similarities of CSF-Lps to plasma HDL, we compared our list of proteins to an aggregated list of previously reported HDL-associated proteins available through the “HDL Proteome Watch” (*3*). The Venn diagram in Fig. 5A shows that more than half of the proteins identified across CSF-Lp populations have not been previously reported in plasma HDL, indicating that CSF-Lps have unique compositional signatures distinct from those in plasma. Though most of the identifications were previously unreported, we found that these proteins only made up ~15% of the total protein abundance (Fig. 5B).

**Fig. 6.**
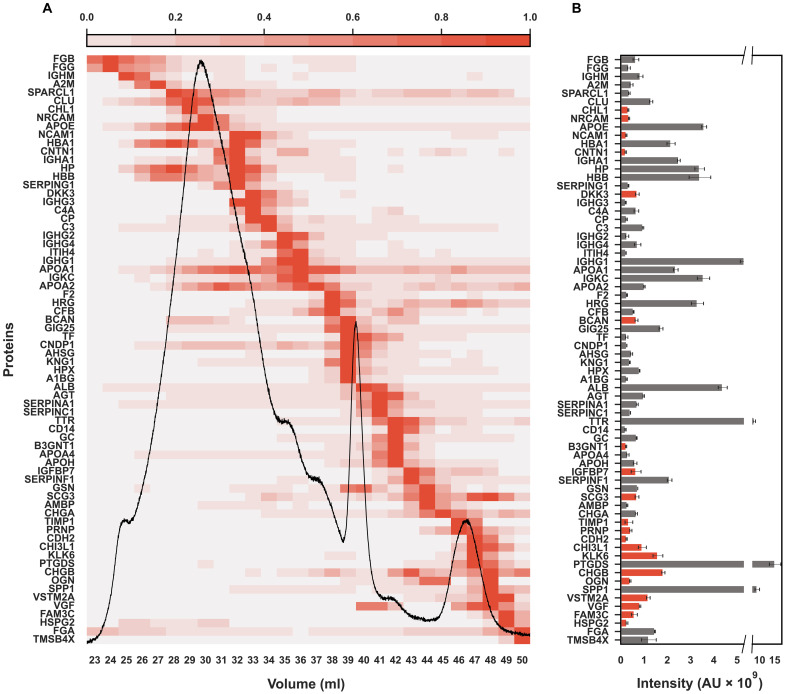
Distribution of top 70 proteins relative to the CSF-Lp distribution obtained by FLP. The total abundance of each protein was determined by summing averaged LFQ intensity of the protein across fractions, and distribution was normalized to the fraction with the highest abundance (1.0). (**A**) Distribution of the 70 most abundant proteins sorted by elution volume from earliest to latest. The black trace represents the phospholipid distribution determined using the FLP. (**B**) The corresponding total abundance of the top 70 most abundant proteins. Gray bars represent proteins previously reported on the HDL Proteome Watch, and red bars represent unique proteins identified in the current study. Bars represent the average of *n* = 3 samples ± SD.

To get a better idea of the relationship between the CSF-Lp proteome and the lipid signal, we took the top 70 most abundant proteins and superimposed the FLP signal to visualize their elution profiles and abundance relative to the lipid trace (Fig. 6). The bulk peak of the particles co-elutes with *APOE*, which was one of the most abundant proteins detected. *APOA1* is also highly abundant but appears most prevalent in particles consistent in size with plasma HDL. We observed that *APOA1* appears to co-elute with *APOA2*, which is found on most HDL containing *APOA1* (*23*, *24*) suggesting that a similarly strong relationship exists between the two proteins on CSF-Lps. We further note that *APOA1* co-elutes with several other proteins reported on plasma HDL including complement factors, serotransferrin, and hemopexin (*2*, *23*). Some of the more abundant unique proteins identified in the study eluted in fractions consistent with very small or poorly lipidated HDL, including kallikrein related peptidase-6 (*KLK6*), secretogranin-1 (*CHGB*) and neurosecretory protein VGF (*VGF*) all shown in red. Prostaglandin D synthase (*PTGDS*) was by far the most abundant protein detected in the study followed by osteopontin (*SPP1*) and transthyretin (*TTR*). A comprehensive heat map of the distribution of all 303 identified proteins across fractions is provided in fig. S6.

### Functional enrichment

To identify potential biomolecular pathways modulated by the CSF-Lps, we performed an enrichment analysis using the Database for Annotation, Visualization and Integrated Discovery (DAVID) on the CSF-Lp proteome. DAVID is a bioinformatic server that identifies biological processes and molecular pathways previously reported to be associated with a given protein. DAVID initially reported 1242 gene ontology (GO) terms for biological processes (GO-BP) enriched from by the 303 identified proteins. We then applied a Bonferroni correction that narrowed the list to 272 significantly enriched GO-BP terms across 290 of our identified proteins. The range in the number of proteins that contributed to a given GO-BP term varied from as low as 5 proteins to a high as 136 proteins with an average association being 42 proteins.

**Fig. 7.**
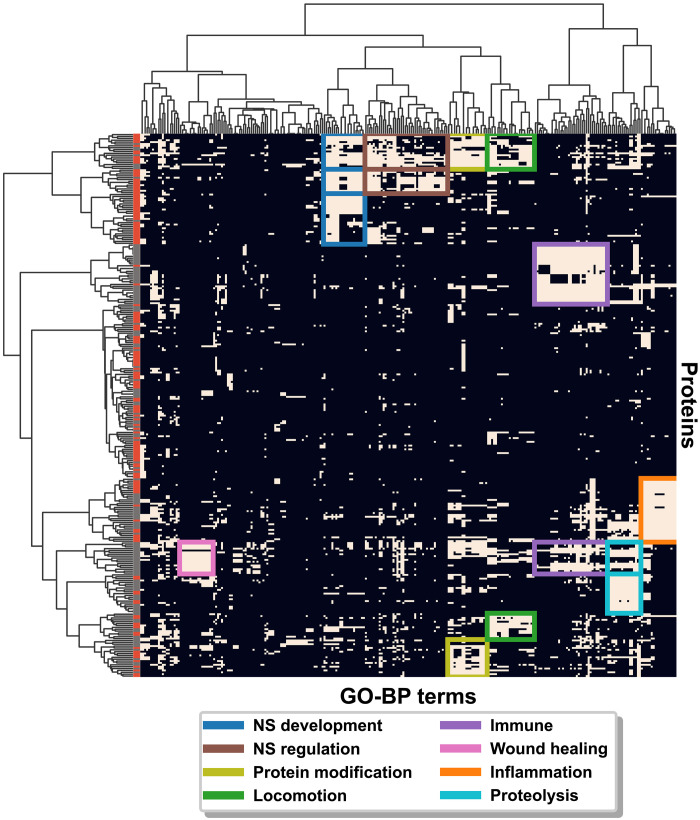
DAVID functional enrichment analysis of the CSF-Lp proteome. Cross-correlation matrix of the relationship between identified proteins (*y* axis) and functional enrichment determined from GO-BP terms using DAVID (*x* axis). Relationships between proteins and GO-BP are indicated as related (tan) or unrelated (black). Hierarchical clustering was performed for the proteins and GO-BP terms independently that are represented by the dendrograms on each axis. Functional clusters are indicated by a colored box, where the color signifies the main category of the cluster’s GO-BP functions. Coloring on the *y* axis indicates whether the protein was found in HDL Proteome Watch Dataset (gray) or the protein was unique to the current study (red). NS, nervous system.

A major challenge with GO enrichment analysis is dealing with semantic similarity that exists within the GO database that often confounds biological interpretation. To better visualize and extract meaningful relationships, we created a functional enrichment matrix between the CSF-Lp proteome and the reported GO-BP terms (Fig. 7). In total, we identified 15 functional clusters in the matrix that span a variety of biological processes including immune response, inflammation, wound healing, proteolysis, and both nervous system development and regulation. Many of these pathways overlap with those previously reported on plasma HDL, which is to be expected given ~50% of the proteome identified in the current study overlaps with the HDL proteome. However, many of the proteins unique to the current study (red on vertical axis) contributed to enrichment of pathways important for CNS function. In total, we identified five functional clusters that generally spanned processes important for either CNS development (blue) or regulation of the development process (brown). Most of the proteins were shared across the processes with the exception of a unique cluster of proteins for CNS development. Further, we observed subgroups of proteins important for protein modification and locomotion, two biological processes that are critical for proper neuron development. Deeper examination showed specific enrichment in proteins that promote neurogenesis and axonogenesis. A detailed list of the individual GO-BP terms and associated proteins is available in table S2.

### Network analysis

**Fig. 8.**
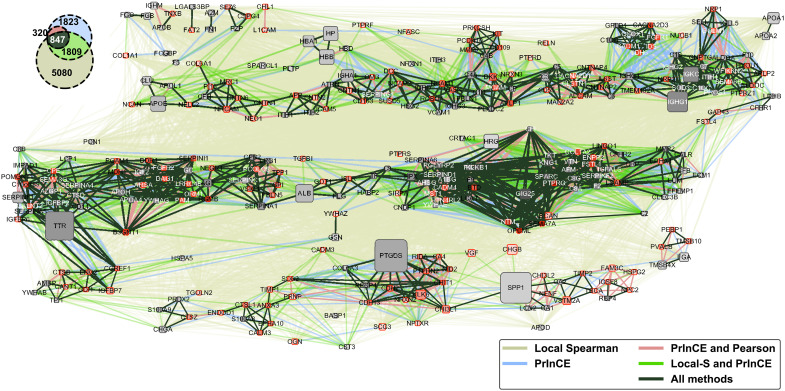
Network analysis of the CSF-Lp proteome. Each node represents a protein identified in the CSF-Lp fractions. The size of the node reflects the total abundance of the protein calculated by summation of LFQ intensity of that protein across fractions. Proteins are generally grouped on the basis of the elution windows from early fractions (top left) to smaller fractions (bottom right) following a serpentine pattern down the rows. Gray nodes represent proteins previously reported on HDL Proteome Watch, and red nodes represent proteins unique to the current study.

The ability to resolve specific combinations of proteins that reside on different CSF-Lps is important for identifying specific particle subspecies that exist such as the TLF population reported in plasma. To identify potential protein clusters on the CSF-Lps, we applied multiple unique computational approaches that investigate the relationship of protein pairs based on (i) previous reports in the literature, (ii) biophysical and biochemical measures such as protein abundance and comigration, and/or (iii) machine learning. From this, we generated the comprehensive protein interaction network shown in Fig. 8. The map is multitiered meaning that the edges, i.e., the lines used to report a positive relationship between two given proteins, are overlayed for the respective approaches. As detailed in Materials and Methods, we ultimately omitted STRING due to lack of previously reported proteomic analyses on human CSF-Lps. A comparison of the common and unique “edges” observed between protein pairs from the Pearson correlation coefficient (PCC), local Spearman correlation coefficient score (Local S-Score), and PrInCE is shown in the Venn diagram in Fig. 8 (inset). As expected, the local Spearman analysis resulted in the most relationships (*n* = 7736) due to the nature of the analysis that essentially collapses 24 individual networks across fractions into a single global map. PrInCE analysis identified the next most relationships (*n* = 2624) with about half of the relationships observed from PrInCE also identified in the Local S-Score. The PCC analysis was the most constraining identifying 1167 relationships, all of which were identified using PrInCE and most of which also in agreement with the Local S-Score.

Next, we aimed to leverage the protein-interaction data from the edges to extract meaningful protein-protein relationship information obtained across the different particles. We focused on only the most robust relationships that were identified across all three computational approaches (847 total edges) and extracted protein “cliques” that are defined as fully interconnected protein subgroups. In total, we identified 157 cliques using edges shared across all three network approaches. Most of the cliques contained 2 to 3 proteins though instances of cliques containing upwards of 10 proteins were observed. Notable cliques identified include *APOA1* and *APOA2*, whose relationship is well established in plasma HDL (*23*, *25*). Similarly, we observed connections between *APOE* and Clusterin (*CLUS/APOJ*), which are thought to be the primary scaffold proteins on CSF-Lps (*26*). A full list of identified cliques shared across all three approaches can be found in table S3.

## DISCUSSION

Arguably the single, biggest impediment to a better understanding of how CSF-Lps modulate CNS health and neurodegenerative disorders is their low concentration in CSF, requiring impractical amounts of sample for in-depth biochemical analysis. Our FLP overcomes this obstacle to resolve previously unreported lipoprotein populations in as little as 350 μl of human CSF. By coupling the FLP with high-sensitivity LC-MS/MS in a single workflow, we reveal that human CSF-Lps exhibit remarkable compositional complexity associating with at least 303 different proteins. Further, our computational and bioinformatic analysis show that similar to plasma HDL, these proteins have reported functions in a variety of important metabolic pathways including the immune response, wound healing, and inflammation. However, we also show that CSF-Lps have compositional features that distinguish them from plasma HDL in that they are enriched in proteins reported to be critical for nervous system development and function. Using a suite of computational tools that leverage patterns within our biophysical data, we show strong evidence that specific clusters of these protein coalesce onto distinct CSF-Lp subspecies of various sizes. These studies strongly support the hypothesis that lipoproteins generated in the CNS play a pleiotropic role in maintaining CNS homeostasis likely through the deployment of unique particle subspecies with distinct compositional signatures that impart specific functions.

Earlier studies on CSF-Lps mostly used preparative ultracentrifugation (*17*, *27*–*29*). While isolation of lipoprotein subclasses based on density has historically served as the “gold standard,” we (*23*) and others (*16*) have demonstrated that this technique notably alters particle composition by stripping off functionally important proteins. Moreover, density separation fails to resolve many of the unique subspecies (*30*) because of physiochemical similarities between the different populations. The major advantage of the gentler SEC isolation procedure used here is that it provides quantifiable information on the size distribution of CSF-Lp subspecies while maintaining the structural integrity of the particles. Consistent with studies that also used SEC to examine CSF-Lps, we report that most CSF-Lps elute in a size range between that of plasma HDL and LDL (*15*, *16*). However, with the increased resolution using three columns in-tandem and added sensitivity of fluorescence, we show that CSF-Lps exhibit far greater size heterogeneity than previously reported.

A deep literature search on CSF-Lp composition and function shows that only 16 proteins have been identified on human CSF-Lps to date. A full list of these proteins can be found on the Brain Lipoprotein Proteome Watch (https://homepages.uc.edu/~davidswm/bLPproteome.html), which we recently launched to serve as a centralized and freely available database to aggregate data from past and future proteomic studies on human CSF-Lps. Of those 16 proteins, only 6 proteins have been reported in three or more studies: *APOE*, *CLUS/APOJ*, *APOA1*, *APOA2*, *APOA4*, and *APOD*. The low number of proteins reported to date likely reflects the aforementioned abundance issues, isolation procedures, and limited immunoassays available at the time. All six proteins were identified in the current study and, consistent with previous reports (*15*–*17*), *APOE*, *CLUS/APOJ*, and *APOA1* were among the proteins of highest abundance. We note that while enriched in specific fractions (Fig. 6), these proteins elute across a broad range of fractions, indicating that they are associated with multiple CSF-Lp populations of various size. This is consistent with the notion that these apolipoproteins serve as dominant organizing “scaffolds” that encapsulate the lipid (*31*, *32*) and typically accounts for the most of the protein detected on a given lipoprotein particle. Our study shows *APOE* mostly co-elutes with the bulk of the lipid also in agreement with previous reports that *APOE* is the primary scaffold protein on CSF-Lps (*26*). Though *APOE* is generated independently in both the periphery and CNS (*33*), it cannot cross the blood-brain barrier (*34*); thus, most of the *APOE*-containing CSF-Lps in this size range are likely generated de novo in the CNS. On the other hand, we detected both *APOA1* and *APOA2*, which are scaffold proteins on plasma HDL. Neither are produced in the CNS and are thought to enter CSF by traversing the blood-brain barrier or via the choroid plexus (*35*). *APOA1* and *APOA2* exhibited almost identical elution patterns and peaked across fractions remarkably consistent with analogous procedures that speciate plasma HDL (*2*). This observation in CSF supports our previous work indicating that *APOA1* and *APOA2* scaffolds are key determinants of particle size (*24*). Whether these plasma-derived protein scaffolds enter the CNS in a fully delipidated state to form particles in the CNS or whether they arrive as poorly lipidated nascent HDLs that mature into spherical form upon arrival is not known. Additional structural studies are required to better understand the role of these scaffold proteins in CSF-Lp biogenesis in the CNS.

Taking advantage of the sensitivity of modern mass spectrometers, our study shows substantial proteomic diversity exists within CSF-Lps. Most of this observed diversity can likely be attributed to “accessory” proteins, which we define as modifying enzymes or cofactors that dock with the particles to impart specific functions (*6*). Docking can be modulated by conformational changes in the scaffold protein (*36*) or directly with the lipid surface (*37*), and multiple accessory proteins can associate with a given subspecies. Most proteins in our study eluted in single Gaussian peaks, suggesting that they prefer to dock with CSF-Lps of specific sizes. As with *APOA1* and *APOA2*, the proteins we identified in CSF that overlap with plasma lipoprotein studies elute in similar size ranges as their plasma counterparts (*2*). For example, complement proteins (*C4A* and *C3*), ceruloplasmin (*CP*), transferrin (*TF*) and kininogen 1 (*KNG1*) all elute in the HDL size range consistent with the study in plasma (*2*). Unexpectedly, we also observed particles in the size range of VLDL/LDL that, like plasma VLDL/LDL, were enriched in fibrinogens and alpha-2-macroglobulin (*A2MG*) (*2*). We also observed *APOB* in these particles but note that it was one of the least abundant proteins identified in the study. We suspect that a combination of the scaffold protein and surface curvature modulate these interactions, which explains the consistency in the observations between the two compartments.

The computational approach coupling functional enrichment pathways with a detailed protein interaction maps strongly suggests that CSF-Lps are a collection of compositionally and functionally unique subspecies. For example, our functional enrichment matrix shows that CSF-Lps are enriched in proteins important for wound healing. Turning to our network map of protein cliques, we see strong relationships between fibrinogen subunits (*FGG* and *FGB*) that play essential roles in blood coagulation and wound repair (*38*). Similarly, we observe edges between several of the complement factors (*C4A-C1R*, *C4A-C4B*, *C3-C5*, etc.) that tightly regulate the innate immune response (*39*), another functional cluster identified in our matrix. We also observed several of the cliques identified on CSF-Lps (e.g., *SERPIND1-CADM4-C9-AHSG-HPX*, *CNTN1-SERPING1-AGHA1*, and *CLUS-APOL1*) overlap with those identified on HDL (*25*). However, our approach used only a single biophysical separation, while the studies on plasma HDL used multiple orthogonal speciation techniques (*25*, *40*), underscoring the power of our computational approach. Though we did not directly test CSF-Lps function, it is important to note that the role of HDL in several of the functional pathways identified in the current study (Fig. 7) has been experimentally validated [reviewed here (*41*)]. Given the importance of these functional pathways in maintaining vascular integrity in the periphery, it makes sense the CSF would contain functional analogs that perform similar tasks in the cerebrovasculature.

**Fig. 9.**
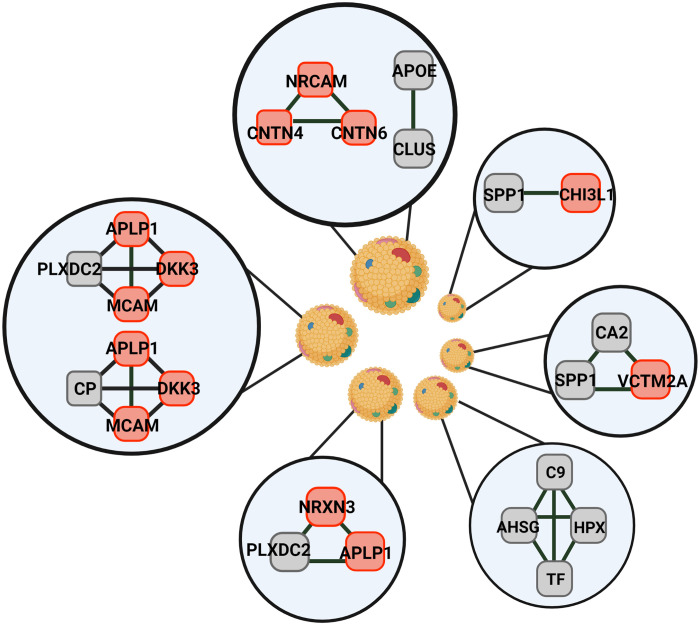
Representation of potential CSF-Lp subspecies. CSF-Lps consist of a constellation of particles of different sizes. Particles within different size classes contain unique protein network hubs consisting of both proteins previously reported on plasma HDL (gray) and proteins unique to populations that exist within the CNS (red).

One of our more notably findings was the enrichment of CSF-Lps in proteins with reported roles in in neurogenesis, maintenance, and function. The network analysis suggests that multiple neuro-specific subspecies exist to carry out these processes that we highlight in the summary Fig. 9. For instance, we identified a clique containing neuronal cell adhesion molecule (*NRCAM*) and contactins 4 and 6 that modulate axon growth and function (*42*–*44*). Lipoproteins have been experimentally demonstrated to enhance axon growth (*45*) though the role of the protein components in this process has yet to be determined. Another interesting clique contains neurexin 3 (*NRXN3*), amyloid precursor-like-protein 1 (*APLP1*), and plexin domain containing 2 (*PLXDC2*). All three of these proteins are known substrates for proteolytic processing by β-secretase 1 (*46*–*48*). Dysregulation of this process can result in production of neurotoxic amyloid beta (Aβ) peptides that form amyloid plaques associated with multiple neurodegenerative disorders. *NRXN3* and *APLP1* are also both reported to regulate synapse formation and function (*49*, *50*), a process also modulated by *APOE* (*51*). Less is known about *PLXDC2* though it is thought to promote cell proliferation (*52*). The analysis identified multiple cliques that contain both Dickkopf-related protein 3 (*DKK3*) and *APLP1*, which have been demonstrated to colocalize in human brain tissue obtained from patients with AD (*53*). Similarly, we identified a strong relationship between serotransferrin and hemopexin (*TF/HPX*), also identified on HDL (*25*) and recently shown in plasma to be associated with altered Aβ metabolism and AD (*54*).

Given that CSF-Lps are reportedly derived primarily from astrocytes (*55*, *56*), the enrichment of proteins important for neuron function suggests CSF-Lps facilitate cross-talk between different cell types in the CNS. We report a distinct cluster of small CSF-Lps punctuated by the presence of secreted phosphoprotein 1 (*SPP1*), a protein up-regulated in activated perivascular macrophages and microglia (*57*). Notably, specific microglia subpopulations have recently been identified that are defined by their expression levels of *SPP1* and *APOE* (*58*). *SPP1* is required for release of opsonins such as *C1Q* (*59*), which tag abnormal synapses for removal by phagocytic microglia. *C1Q*, which can complex with *APOE* (*60*), was identified in several cliques in the current study. The partitioning of *SPP1* and *C1Q* into different cliques suggests that multiple CSF-Lp subspecies could be acting in concert to tightly regulate synaptic pruning and temper neuroinflammation. The role of CSF-Lps in this process warrants further investigation given the impact of hyperactive microglia and lipids in dysregulated synapse phagocytosis in the development of AD (*61*).

We recognize that our proteomics analysis shows considerable overlap with compositional signatures inherent to plasma lipoproteins, raising a concern of plasma contamination. For instance, we observed trace levels of *APOB* in our larger-sized fractions. Dogma states that *APOB* is not present in CSF as it is not thought to cross the blood-brain barrier (*62*). On the other hand, more recent studies are emerging that report *APOB* in CSF (*63*, *64*). Of the proteins identified in our study, *APOB* was vanishingly small barely meeting our thresholds for reporting. We also observed the hemoglobin-haptoglobin complex (*HBB-HBA-HP*) in our network analysis. This is a well-recognized complex in blood; however, we note the concentrations of these proteins, and their abundance ratios were not consistent with those measured in plasma. A recent study reported using *HBB*, *HBA*, *HBD*, *CAH1*, and *CATA* as a combinatorial biomarker for blood contamination (*65*). Using the scale provided by authors for quantifying contamination, our data is consistent with ~0.01% contamination, which is well below the 0.1% lower limit that the authors suggest influence data interpretation. Thus, while we recognize that blood contamination may exist, we conclude that its level has minimal impact on our results.

We also recognize that the current study has limitations. For one, any lipid-containing particle likely binds to the LRA that includes lipoproteins, microparticles, and extracellular vesicles. We make no attempt to distinguish between these different particle types; we define lipoproteins in the simplest sense as a lipid-protein complex. On the other hand, we cannot rule out that some of the proteins detected in the analysis might bind to the LRA independent of presence of lipid. This was previously tested in plasma (*2*) where several of our identified proteins were reported as potential nonspecific binders. However, we note that most of the proteins reported on that list have since been documented to associate with lipids including the fibrinogens (*66*) and serum albumin (*67*) speaking to the difficulty in distinguishing between specific and nonspecific interactions. All of the proteins on that list have been independently detected in at least three unique studies on HDL as reported on the HDL Proteome Watch. This speaks to the power of maintaining a database that aggregates studies on lipoproteins using a variety of alternative biophysical isolation techniques. The Brain Lipoprotein Proteome Watch will serve an equally important role.

In summary, we present a pipeline for deep molecular phenotyping of lipoproteins in small volumes of human CSF. Using this pipeline, we reveal that CSF contains a heterogeneous population of compositionally unique lipoproteins that, like plasma HDL, are enriched in proteins with functions beyond simple lipid transport. Collectively, our observations strongly support a role for CSF-Lp subspecies in modulating molecular processes critical for information processing and cognition. Given that quantification of shifts in plasma lipoprotein size has provided profound insights into the etiology of multiple metabolic disorders (*20*, *21*, *68*), future work will focus on molecular profiling of CSF-Lps to identify functionally relevant subspecies in individuals suffering from neurodegenerative disorders. Further, there is a need for the development of assays to validate the functional role of different CSF-Lp subspecies in CNS metabolism.

## MATERIALS AND METHODS

### Human sample collection and storage

We obtained human plasma from healthy donors from the Hoxworth Blood Blank in Cincinnati, OH. Trained phlebotomists collected blood under an approved Institutional Review Board protocol from the University of Cincinnati. All samples were deidentified. Upon receipt, we added 5% sucrose (v/v) and stored plasma at −80°C until ready for use. We purchased pooled human CSF from Medix Biochemica (catalog no. 991-19-P) which we stored at −80°C until ready for use. For testing the impact of flash-freezing CSF, we acquired fresh samples from the laboratory of H. Yassine at University of Southern California that were shipped overnight on wet ice. Upon arrival, we aliquoted the sample for two experiments. We stored the “nonfrozen” sample at 4°C for analysis within 72 hours of arrival. We flash-froze the second aliquot in liquid N_2_ and stored at −80°C until ready for analysis. This work abides by the Declaration of Helsinki principles, and thus, donors provided informed consent before sample collection.

### Lipoprotein labeling

Label consisted of a free rhodamine (MilliporeSigma, catalog no. 83689) and 18:1 Liss Rhod PE (Avanti, 810150). Note: Liss Rhod PE contains its fluorescent group on the phospholipid (PL) headgroup, not on the fatty acyl chains, to minimize chances of membrane perturbations upon labeling of lipoproteins. We brought the free rhodamine to a final stock concentration of 0.0025 mg/ml with dimethyl sulfoxide (DMSO). We dried the Liss Rhod PE stock (1 mg/ml in chloroform) under nitrogen gas and placed in a vacuum for 30 min to ensure complete removal of chloroform. Once dry, we resuspended the Liss Rhod PE in the free rhodamine DMSO stock to a final concentration of 0.01 mg/ml and incubated the mixture at 37°C for 15 min with intermittent vortexing. We aliquoted 15 μl of the fluorescent label into glass test tubes that we purged under nitrogen gas, sealed, and stored at −20°C until use. For labeling lipoproteins, we thawed the label at room temperature and added the plasma or CSF directly to the tube containing the label. Most studies used either 500 μl of plasma or 1 ml of CSF for labeling and profiling. We added plasma directly unless otherwise noted. We concentrated CSF using Amicon Ultra-4 centrifugal filters (molecular weight cutoff = 3500) at 2000*g* at room temperature and brought to a final volume of 400 μl with STB [150 mM NaCl, 10 mM tris-HCl, 1 mM EDTA, and 0.02% azide (pH = 8.2)] and added to the label. For experiments using unconcentrated CSF, we added 400 μl directly to the label. After addition of the biofluid to the label, we lightly vortexed the sample and incubated for 16 hours at room temperature in the dark.

### Fluorescent lipoprotein profiling

We sterile-filtered the samples after incubation and applied 350 μl to the FLP for fractionation. We achieved separation using high-resolution SEC that consisted of three Superdex 200 increase columns (Cytiva) in-tandem (*2*) operating at a flow rate of 0.3 ml/min in STB. We monitored the lipoprotein distribution in real time using a Dionex Ultimate 300 (Thermo Fisher Scientific) equipped with a flow cell integrated between the last Superdex columns and the fraction collector. The Dionex acquired signal by sampling at 2 Hz with an excitation wavelength of 560 nm and an emission wavelength of 583 nm. We obtained the plasma signal at a sensitivity setting of 1 and CSF signal at a sensitivity setting of 8. We eluted samples with five single column volumes of buffer (~120 ml) and collected 1-ml fractions that we sealed and stored at 4°C until ready for analysis. For plasma, we determined phospholipid content enzymatically (Phospholipids C, Wako) on individual fractions as previously described (*2*).

### Preparation for analysis by LC-MS/MS

We isolated lipoproteins from lipid-free molecules in the CSF fractions using LRA as described (*2*). We added 150 μl of LRA in STB (10 mg/ml) to each well containing the entire 1-ml volume of fractionated CSF, resealed the plates, and placed them on a rotator for 1 hour at room temperature. We performed a short spin on the plates to capture all of the material at the bottom of the well, remixed the sample through gentle pipetting, and transferred it to a 96-well filter plate. We removed buffer containing free protein using a vacuum manifold; i.e., the resin containing the lipoproteins was retained in the filter plate. We washed the LRA-bound lipoproteins three times with 150 μl of 50 mM ammonium bicarbonate (AB) to desalt the sample and remove loosely bound material. We resuspended samples in 100 μl of AB containing 1 μg of trypsin to digest proteins on the bound lipoproteins in the wells overnight at 37°C on an orbital shaker. We collected the tryptic peptides into clean 96-well plates placed underneath the filter plate using centrifugation. We reduced disulfide bonds in the tryptic peptides by the addition of dithiothreitol to a final concentration of 10 mM and incubating for 30 min at 42°C. We removed the plates and carbamidomethylated the cysteine residues on the peptides by adding iodoacetamide to a final concentration of 40 mM and incubating at room temperature for 30 min in the dark. We transferred the peptides to washed polymerase chain reaction tubes, took them to dryness, and stored them at −20°C until ready for LC-MS/MS analysis.

### Proteomic profiling of CSF-Lps by LC-MS/MS

We solubilized digested peptides in 200-μl water and loaded them into an HLB 96-well μElution plate (Waters, Milford, MA) for sample cleanup. We washed the samples twice with 200-μl 0.1% trifluoroacetic acid in water and eluted bound peptides from the resin using 100-μl 80% acetonitrile and 0.1% formic acid in water. We dried the peptides down and reconstituted in 1% acetonitrile and 0.1% formic acid in water. We performed a capillary liquid chromatography–electron spray ionization–mass spectrometry analysis using an IntegraFrit capillary C18 trapping column (Waters XBridge BEH C18, 5 μm, 0.1 mm by 40 mm), a capillary analytical C18 column that we packed in-house (Waters XBridge BEH C18, 5 μm, 0.075 mm by 250 mm), and an Orbitrap Fusion Tribrid mass spectrometer (Thermo Electron, Bremen, Germany). In each experiment, we introduced digested peptides onto the trapping column where they were desalted for 10 min at 2 μl/min in 98% solvent A (0.1% formic acid in water). We then directed the effluent from the trapping column to the analytical column at a flow rate of 0.3 μl/min. We eluted bound peptides from the analytical column using a linear gradient from 2 to 35% solvent B (0.1% formic acid in acetonitrile) over 60 min and 35 to 50% solvent B over 5 min. We washed the column for 10 min at 80% B and re-equilibrated at 98% A for 15 min between runs. We introduced peptides into the mass spectrometer through electrospray ionization. We operated the instrument in the data-dependent mode to acquire a full MS scan [400 to 2000 mass/charge ratio (*m*/*z*)] and subsequent MS/MS scans of precursor ions selected on the basis of their intensity. We acquired MS1 scans in the Orbitrap with a resolution set at 120,000 and MS/MS scans in the ion trap with an isolation width of 1.6 *m*/*z*, automatic gain control target of 1.0 × 10^4^, and maximum injection time of 35 ms. We performed the higher-energy collisional dissociation of peptides at 25% normalized collision energy.

### Proteomic data analysis

The workflow for the identification of proteins can be found in fig. S7. We searched the Thermo instrument (.raw) files using MaxQuant (v2.0.1) (*69*) to identify proteins and perform LFQ analysis. We analzyed the experimental spectra within the Raw files against the UniProtKP knowledgebase for *Homo sapiens* (release 2021_03, 20,371 entries). We constrained our searches to tryptic digestion with a maximum of four missed cleavages. Variable modifications included N-terminal acetylation (42.0106 Da) and oxidation (15.9949 Da), and fixed modifications were limited to carbamidomethylation (57.0215 Da). We set parent and fragment ion mass tolerances to 20 ppm and 0.5 Da, respectively. In total, we identified 5649 peptides that corresponded with 873 protein groups (table S1). Though fractions 23 to 60 were analyzed by LC-MS/MS, we limited the data reported to fractions 23 to 50 corresponding to lipid signal on the FLP. We constrained the list of identified proteins to those that (i) contain at least three unique peptides and (ii) appeared in the same fraction across all three replicates. Further, we removed 10 proteins identified in a decoy search against the reversed sequences of the UniProtKP knowledgebase and an additional 21 proteins classified as protein contaminants (e.g., Keratins), narrowing the final list to 303 proteins (table S1).

### CSF-Lp functional enrichment

We performed a DAVID functional enrichment analysis (*70*) on the library of proteins identified across all the CSF-Lp fractions with a focus only on GO-BP terms. We applied a Bonferroni multiple comparison correction to identified GO terms with a Bonferroni <0.01 considered statistically significant. Next, we generated a binary matrix with the statistically significant proteins represented in rows and GO-BP functions represented in columns. We set a related binary relationship between protein and GO-BP to 1 (tan) and unrelated to −1 (black). We then organized the respective proteins and GO-BP terms into dendrograms using SciPy interdependent hierarchical clustering. Thus, branches on the dendrogram for GO-BP functions are organized on the basis of terms that share multiple proteins, while branches on the dendrogram for proteins are organized on the basis of terms that share similar GO terms. For example, two different GO terms that share 8 of 10 proteins will be grouped much closer together compared to two GO terms that share only 1 or no proteins. Ten subgroups were generated across the respective dendrograms to generate a 10 × 10 overlay of 100 potential clusters (fig. S8A). A functional cluster was defined as a block that contained >50% correspondence between the protein and GO-BP terms (fig. S8B).

### CSF-Lp network analysis

We investigated potential protein-protein interactions using four independent network approaches. First, we applied a STRING network analysis (*71*) to query the 303 final proteins from the CSF-Lp master list with a threshold of >0.85 to identify only high-confidence protein-protein interactions. Second, we generated a PCC based on the co-elution pattern of proteins across fractions. Briefly, we normalized individual proteins by the fraction in which that protein exhibited maximal LFQ intensity to scale abundance across fractions between 0 (fractions with no protein) and 1 (fraction with the highest amount of protein X). We then calculated a PCC for every possible protein pair (*n* = 45,753) across the entire CSF-Lp fractionation window. We considered protein pairs with a PCC significance value >0.85 to be related. Third, we generated a local S-Score as previously described (*25*). Briefly, we normalized protein abundance across fractions as with the PCC; however, we calculated a local S-score for each fraction across a sliding window of the given fraction ±2 fractions (*n* = 5 total fractions). This resulted in 24 individual local networks, and pairs within each network were only included if the local S-score > 0.85. For comparison, we collapsed the 24 individual networks into a single global network. For the final approach, we used a PrInCE analysis on the CSF-Lp proteome list using Comprehensive Resource of Mammalian Protein Complexes (CORUM) as our reference database (*72*). We considered a threshold >0.85 to be related. To evaluate the agreement between the different approaches, we applied a Fisher’s exact test. The odds ratio, which measures the strength of association between different methods (the higher the odds ratio, the stronger the agreement), is shown in table S4. An odds ratio >1.0 and corresponding *P* value <0.05 indicate a statistical significant association between the two methods. Though STRING met these thresholds, we found it to be a clear outlier from the other approaches likely due to the unique data generated in the study; i.e., there are essentially no other reported proteomic analyses on human CSF-Lps on which STRING heavily relies. Thus, we opted to omit STRING from additional network analyses and focus on the data-driven computational approaches. While a single threshold of 0.85 may not be directly comparable across methods, we note that it represents a conservative interpretation for each approach. For all approaches, we generated a potential interaction network using proteins as nodes and edges between the proteins to delineate potential protein-protein interactions. For each network, we determined the maximal cliques that define the interprotein relationships using Python NetworkX algorithm.
