# Supplementary material for: Human cerebrospinal fluid contains diverse lipoprotein subspecies enriched in proteins implicated in central nervous system health
Source: Sci Adv. 2023 Aug 30;9(35):eadi5571. doi: 10.1126/sciadv.adi5571 (PMC10468133; doi:10.1126/sciadv.adi5571)
Supplement: Supplementary file 1 — Figs. S1 to S8 Legends for tables S1 to S3 Table S4 [file sciadv.adi5571_sm.pdf]

Science Advances
AAAS

Supplementary Materials for

Human cerebrospinal fluid contains diverse lipoprotein subspecies enriched in proteins implicated in central nervous system health

Nathaniel J. Merrill *et al.*

Corresponding author: John T. Melchior, john.melchior@pnnl.gov

Sci. Adv. **9**, eadi5571 (2023)
DOI: 10.1126/sciadv.adi5571

The PDF file includes:

Figs. S1 to S8
Legends for tables S1 to S3
Table S4

Other Supplementary Material for this manuscript includes the following:

Tables S1 to S3

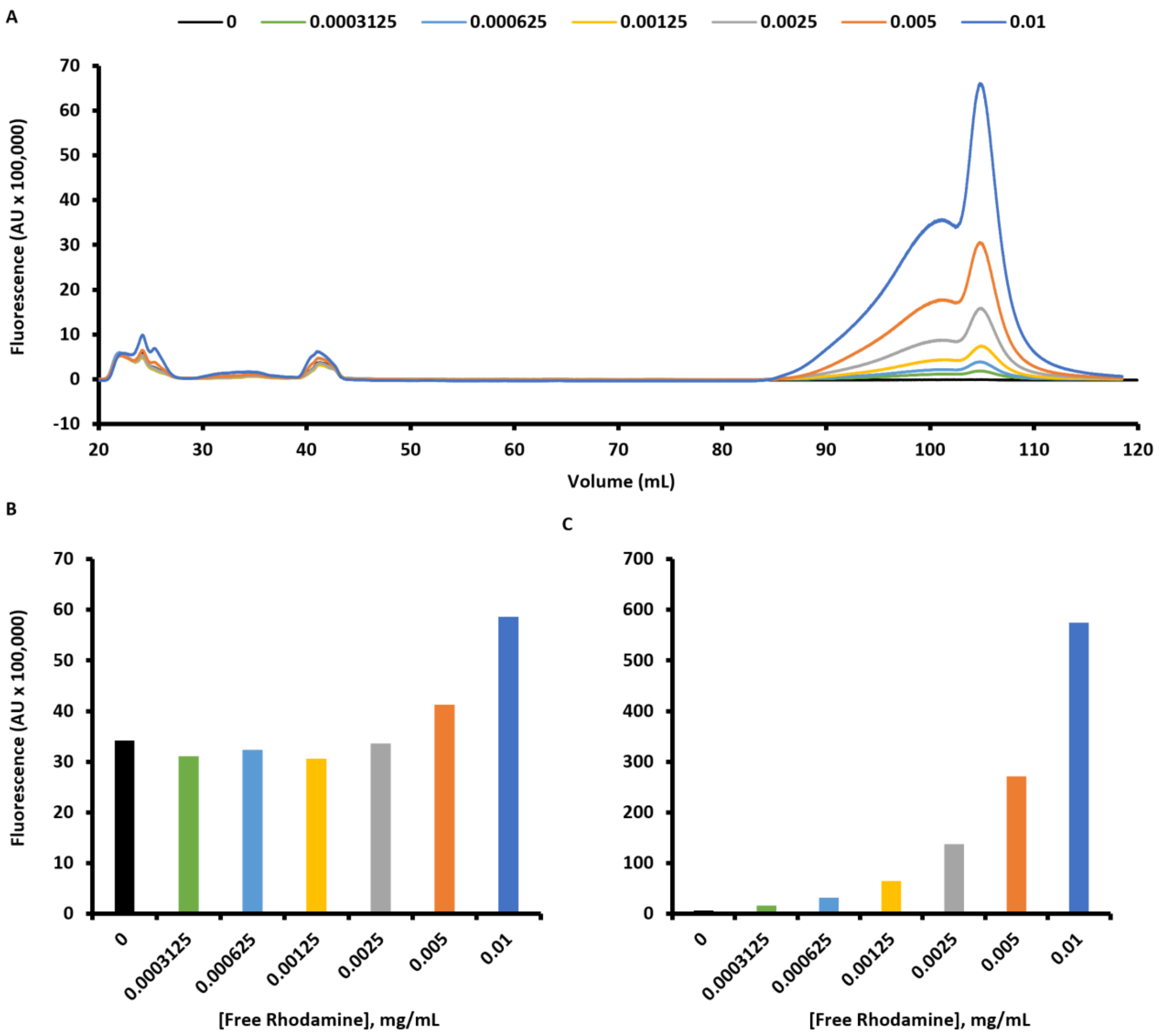

Fig. S1. Optimization of free rhodamine labeling control.

Plasma was incubated with varying concentrations of free rhodamine and applied to the fluorescent lipoprotein profiler (FLP) to monitor signal intensity. **Panel A:** The distribution of fluorescent signal in plasma. **Panel B:** Signal intensity calculations for area under the curve spanning elution volume 20-44 mL. **Panel C:** Signal intensity calculations for area under the curve spanning elution volumes 80-120 mL.

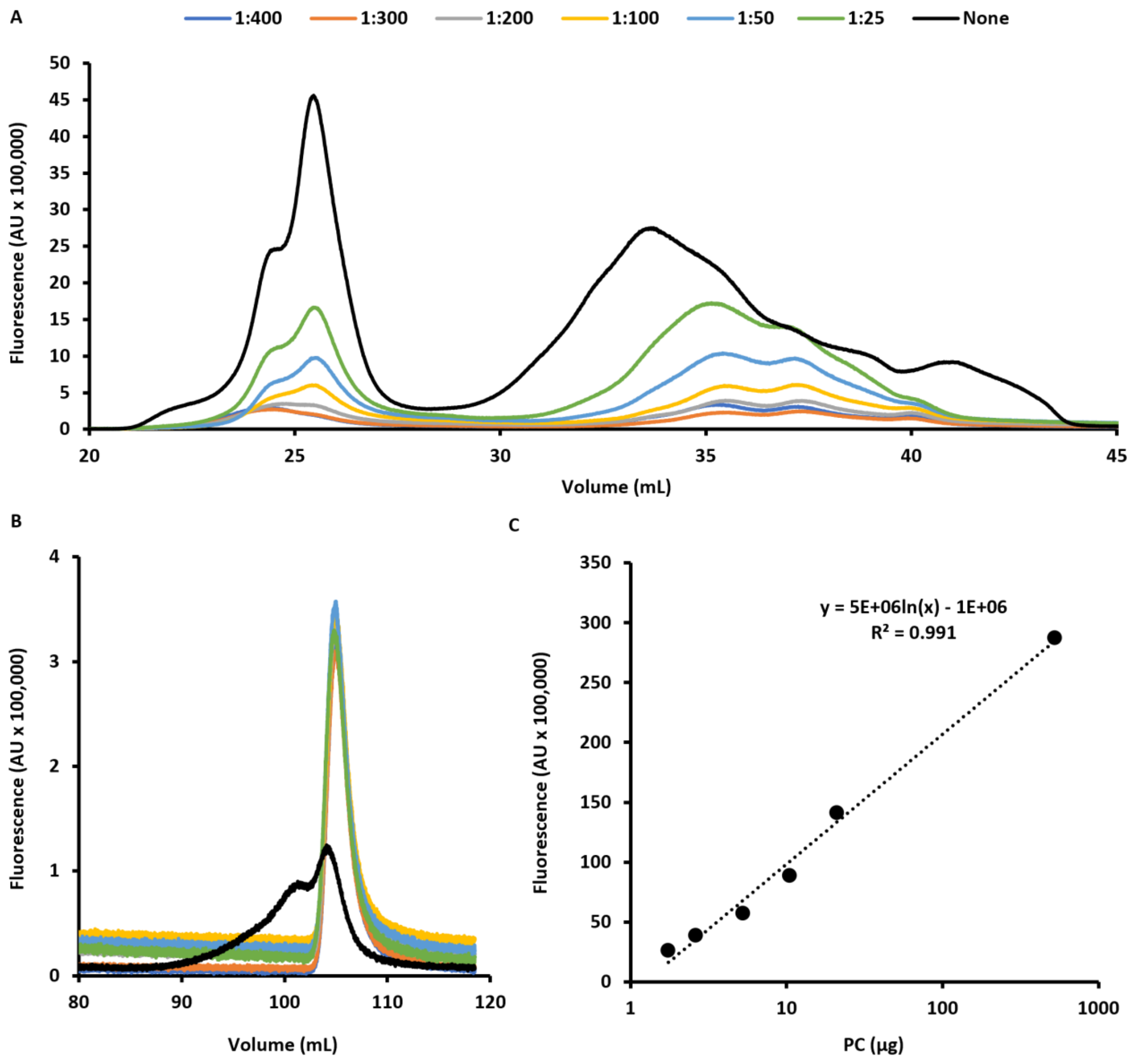

Fig. S2. Relationship between fluorescent signal area under the curve and phospholipid mass.

Plasma was diluted with **standard tris buffer (**STB**)** and labeled as described. **Panel A:** The distribution of fluorescent signal in the diluted plasma obtained from the FLP. **Panel B:** The distribution of fluorescent signal in the diluted plasma for elution volume 80-120 mL consistent with the free rhodamine. **Panel C:** Relationship between phospholipid mass in plasma applied to the FLP with fluorescent signal intensity calculated as area under the curve spanning elution volumes 20-44 mL.

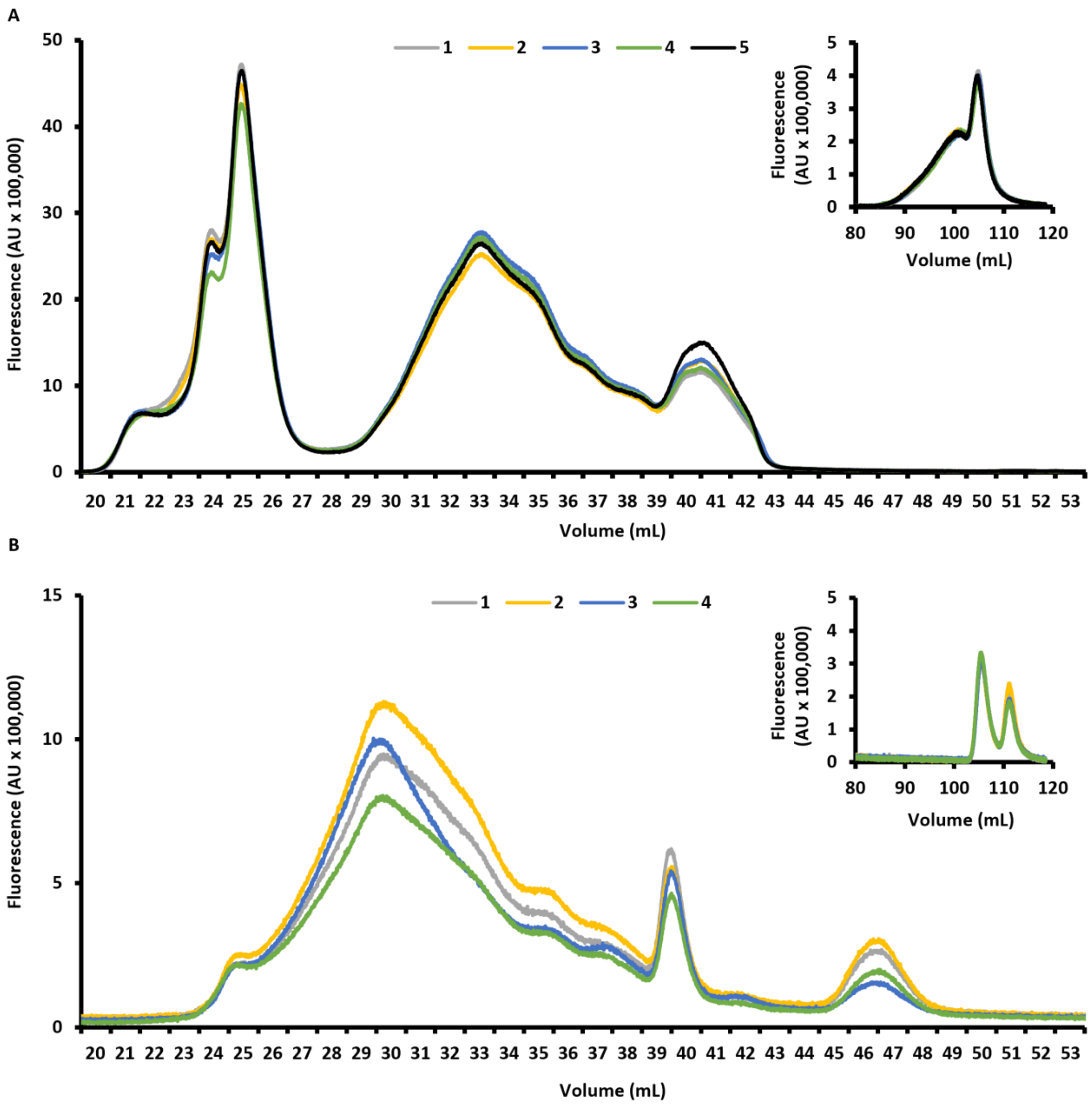

Fig. S3. Reproducibility of labeling in human plasma and CSF.

Panel A: Signal distribution of labeled human plasma lipoproteins (N = 5 replicates) monitored using the FLP with a signal sensitivity setting at 1. **Panel B:** Signal distribution of labeled human CSF (N=4 replicates) monitored using the FLP with a signal sensitivity setting at 10. The inset in each figure shows signal distribution for the free rhodamine loading control.

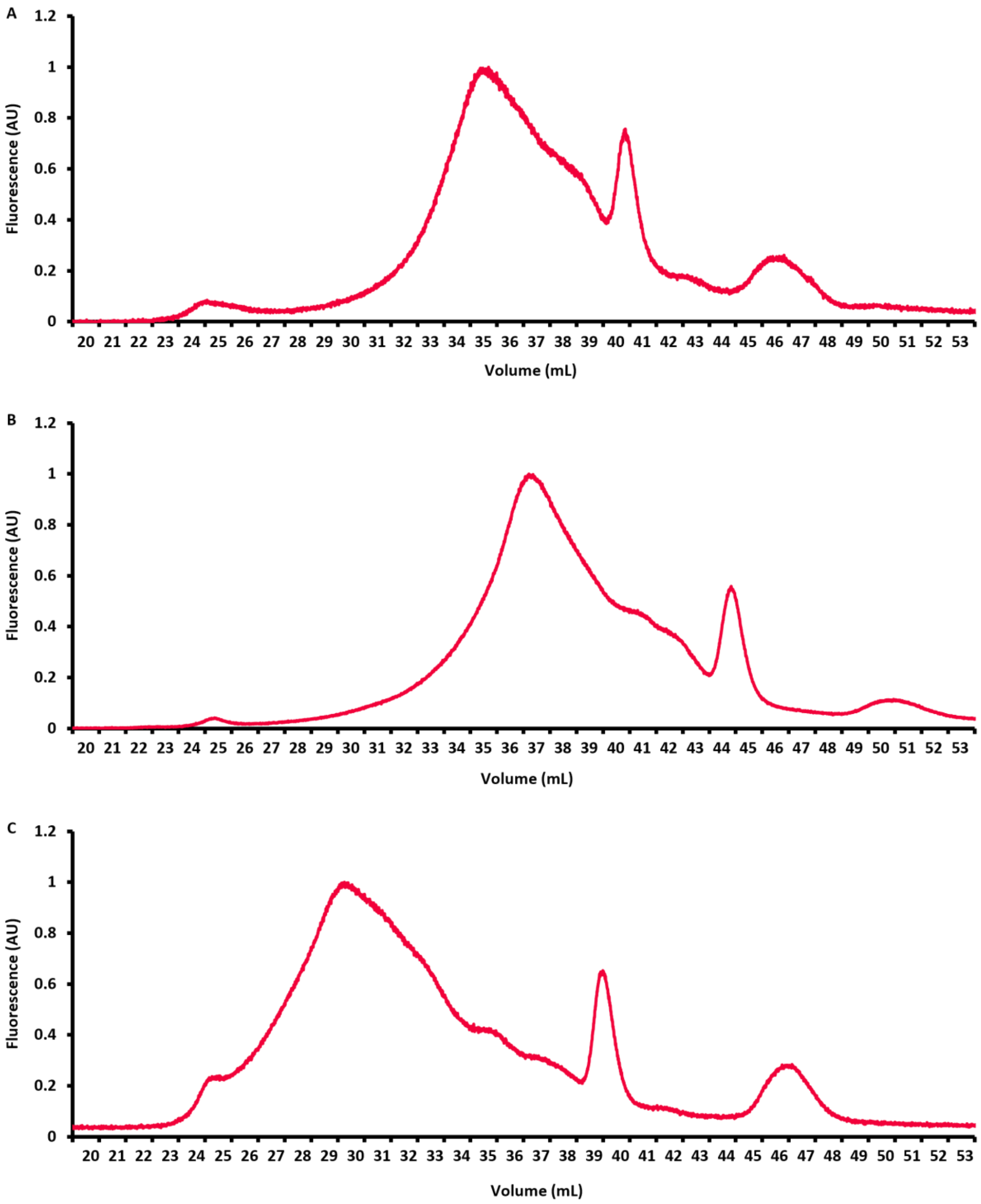

Fig. S4. Distribution of CSF-Lps in human CSF across different SEC setups.

CSF-Lps were labeled and applied to the FLP containing different SEC column setups. **Panel A:** The distribution of CSF-Lps observed when applied to two superose 6 columns followed by a single superdex 75 column in tandem. **Panel B:** The distribution of CSF-Lps when applied to two superose 6 columns followed by a single superdex 200 column in tandem. **Panel C:** The distribution of CSF-Lps when applied to three superdex 200 columns in tandem.

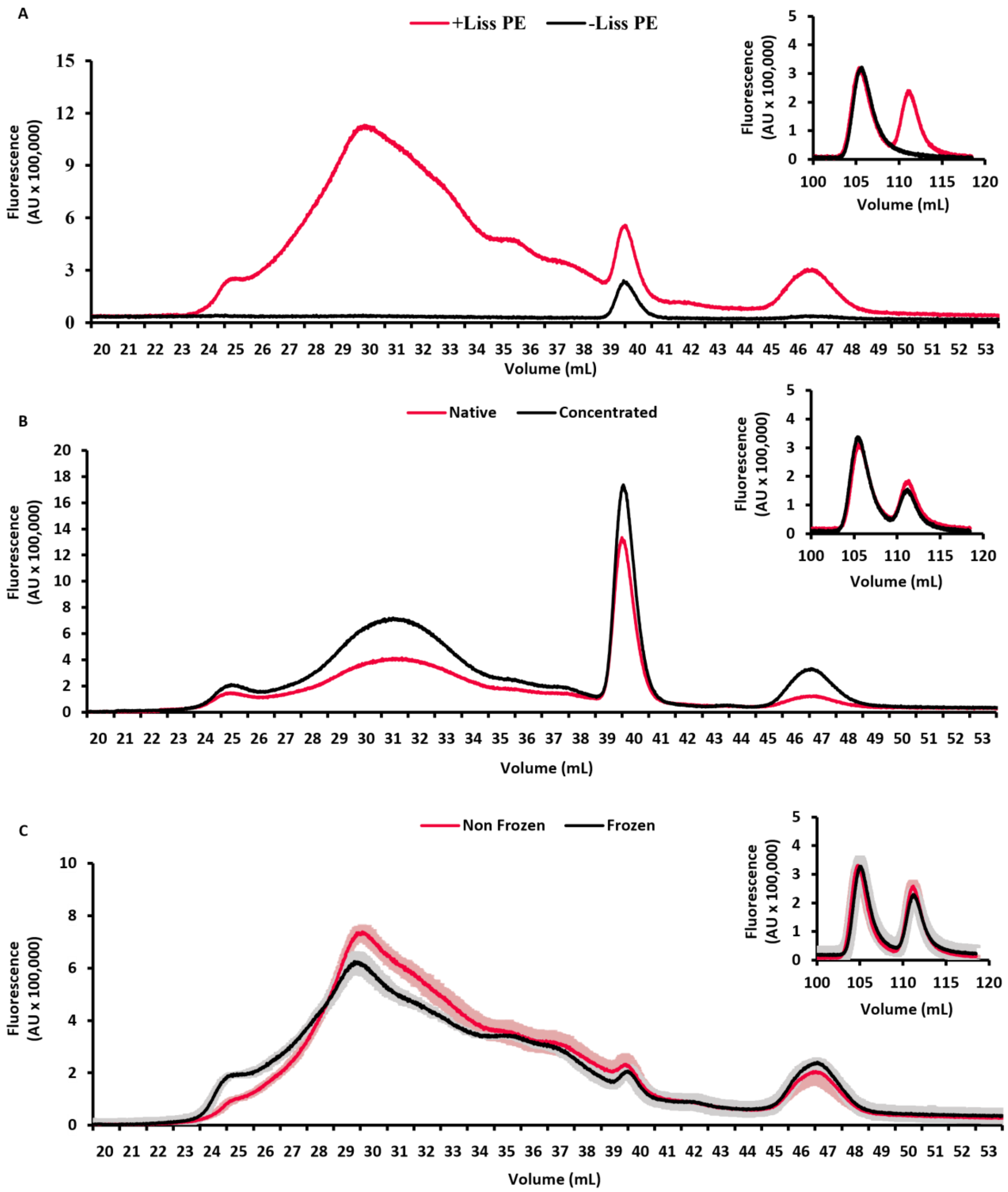

Fig. S5. The impact of biochemical manipulations on the distribution of human CSF-Lps. **Panel A:** Autofluorescence of human CSF molecules. 1 mL of pooled CSF (Lee Biosciences) was concentrated and labeled with either both Liss-PE and free rhodamine or free rhodamine alone and signal distribution was analyzed using the FLP. **Panel B:** Concentration effects on human CSF. Either 400 µL of neat CSF (black) or 1 mL of CSF concentrated to 400 µL (green) was labeled and signal distribution was analyzed using the FLP. Experiments were performed on

a different lot of CSF (Lee Biosciences) than Panel A. **Panel C:** Freshly collected human CSF was obtained and shipped overnight from Dr. Hussein Yassine’s laboratory at University of Southern California. Upon arrival sample was stored at 4 °C. Sample was either immediately labeled and signal distribution analyzed by the FLP (non-frozen) or flash frozen, thawed, labeled and analyzed by the FLP. The distribution of fresh CSF-Lps after collection without freezing (red) and a single freeze thaw cycle (black) is shown. Light areas represent the standard deviation between N=2 samples each.

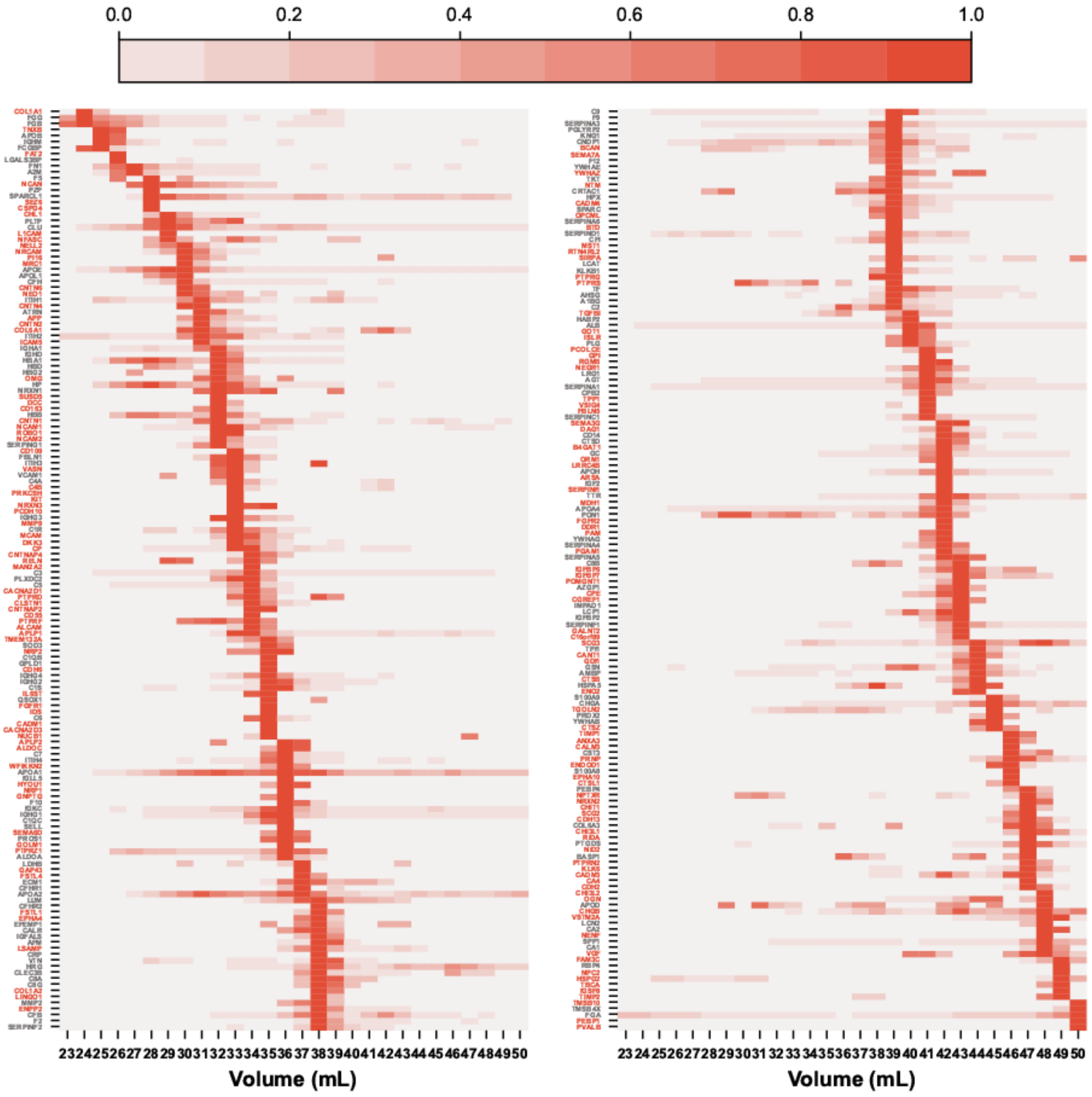

Fig. S6. Distribution of proteins on CSF-Lps isolated across fractions.

The total abundance of each protein was determined by summing averaged LFQ intensity of the protein across fractions and distribution was normalized to the fraction with the highest abundance (1.0). Distribution is sorted by elution volume from earliest to latest. Column two is a continuation of column 1. Protein names in red represent the unique proteins identified in the current study.

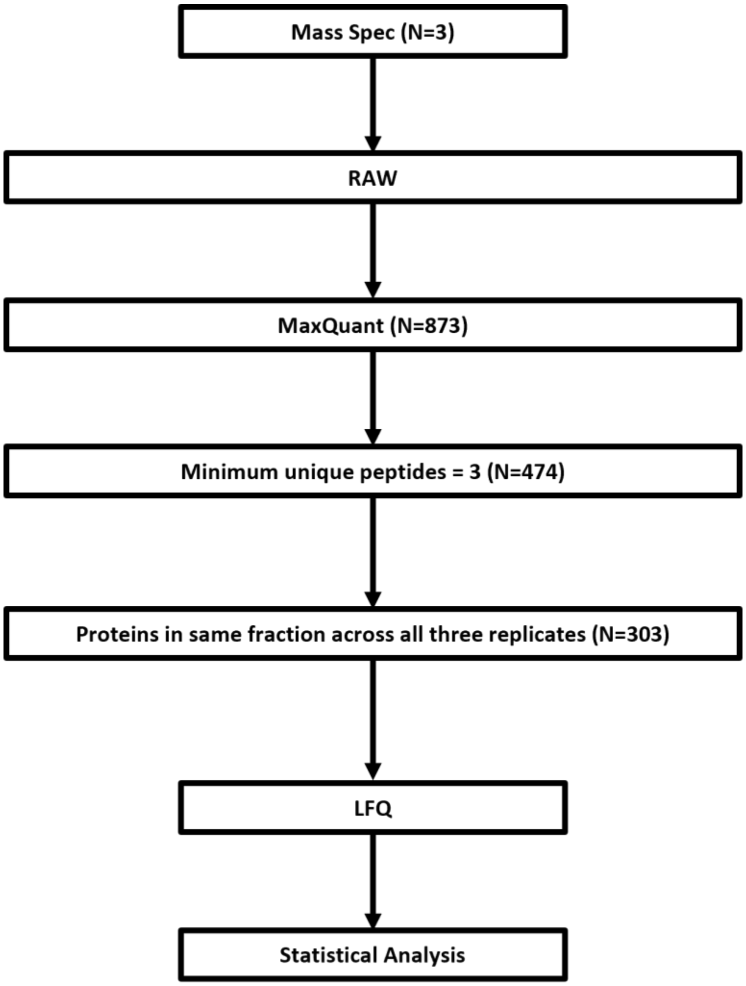

Fig. S7. Proteomic data analysis workflow.

Proteomics analysis was performed on lipoproteins isolated from fractionated human CSF. Thermo .raw files were imported into MaxQuant for LFQ analysis and protein identifications were constrained to only experimentally robust proteins identified using LC-MS/MS.

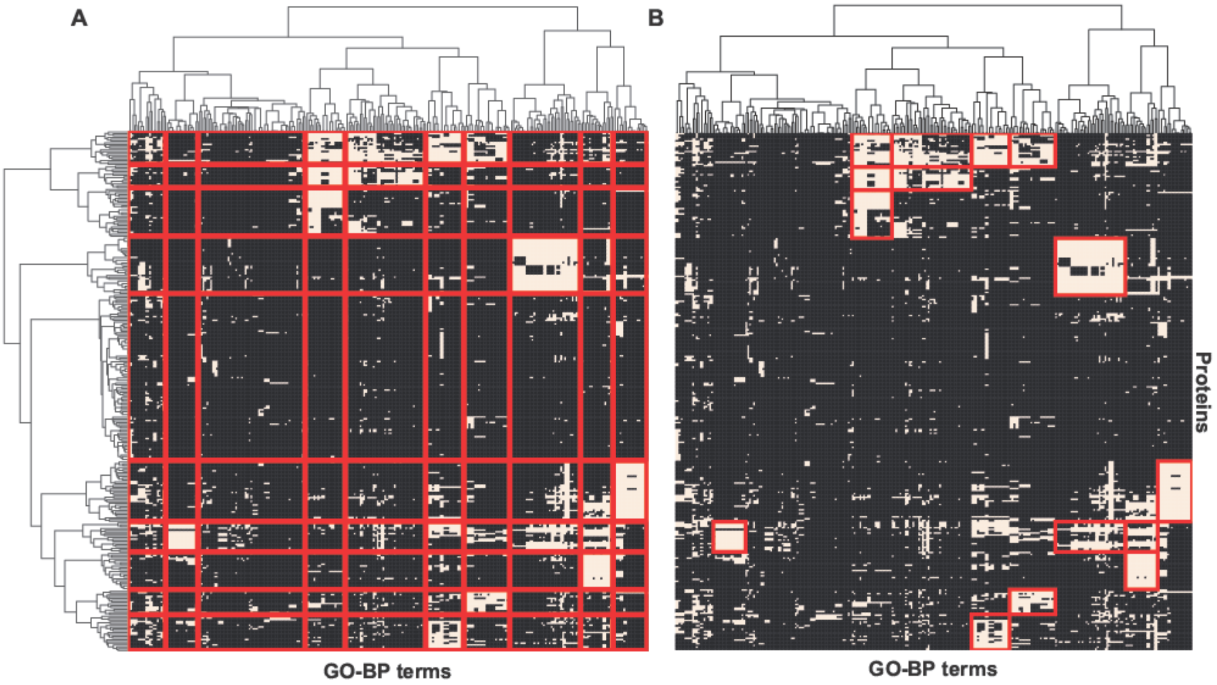

Fig. S8. CSF-Lp Functional Enrichment Analysis.

Cross-correlation matrix of the relationship between identified proteins (y-axis) and functional enrichment determined from Gene Ontology Biological Processes (GO-BP) terms using DAVID (x-axis). Relationships between proteins and GO-BP are indicated as related (tan) or unrelated (black). DAVID functional enrichment analysis was used to identify significantly enriched gene ontology biological process (GO-BP) terms for the CSF-Lps. Proteins and GO-BP terms were independently clustered using SciPy hierarchical clustering, resulting in a binary matrix with proteins as rows and GO-BP functions as columns. Relationships between proteins and GO-BP were indicated as related (tan) or unrelated (black). **Panel A:** The 10x10 overlay of 100 potential clusters. **Panel B:** Final determination of a functional cluster was defined based on blocks with greater than 50% correspondence between proteins and GO-BP terms.

Table S1. Detected proteins on lipoproteins isolated from fractionated human CSF.

Proteins were identified and quantified using MaxQuant as described in *Methods*. Colored cells represent the final 303 proteins reported. Proteins in grey have previously been reported on the HDL proteome watch list and proteins in red are unique to the current study. Fraction number is shown in Row 1 with corresponding LFQ intensities for each protein. LFQ intensities are the averaged over three runs.

Table S2. Detailed list of proteins and associated GO-BP terms in the functional enrichment matrix.

Table S3. Protein cliques identified common to all three bioinformatic analyses.

Proteins relationships were investigated using a Pearson Correlation Coefficient, PrInCE, or Local S-Score. Related proteins identified using all three methods are reported with total number of proteins within each clique. Gray cells represent proteins previously reported on HDL Proteome Watch and red cells represent proteins unique to the current study.

Table S4. Detailed list of proteins and associated GO-BP terms in the functional enrichment matrix.

A Fisher Exact test was used to evaluate the association between the different methods. Infinite means all values were shared between the two methods.

Methods Compared	Odds Ratio	p-value
String-PCC	2.52	3.57E-06
String-PrInCE	1.93	1.04E-05
String-Local S	2.79	2.02E-27
PCC-PrInCE	Infinite	0
PCC-Local S	14.48	0
PrInCE-Local S	10.00	0
